# Fledgling Sex Ratio Is Determined by Egg Loss, Hatching Order, Nestling Mortality, and Inter‐Annual Food Fluctuations for Boreal Owls, *Aegolius funereus*

**DOI:** 10.1002/ece3.71001

**Published:** 2025-03-13

**Authors:** Markéta Zárybnická, Lucie Brejšková, Karolina Mahlerová, Karel Šťastný, Richard Ševčík, Fernando Riera, Wesley M. Hochachka

**Affiliations:** ^1^ Department of Ecology, Faculty of Environmental Sciences Czech University of Life Sciences Prague Prague Czech Republic; ^2^ Institute of Experimental Botany, Academy of Sciences of the Czech Republic Prague Czech Republic; ^3^ Cornell Lab of Ornithology New York New York USA

**Keywords:** food fluctuations, hatching order, inter‐annual variation, nestling mortality, seasonal variation, sex ratio, sexually‐size dimorphism, Tengmalm's Owl

## Abstract

Extensive research has been conducted to explore adaptive variation in offspring sex ratios, focusing on birds as a model group. However, studies to date have not been comprehensive in scope, limiting our understanding of whether there is substantial within‐ or among‐year variation in offspring sex ratios, which environmental conditions and mechanisms are associated with this variation, and when during a nesting attempt the fledgling sex ratio is largely determined. To address these gaps, we analyzed our 18‐year dataset from 542 sexually size‐dimorphic Boreal Owl ( *Aegolius funereus* ) offspring in 140 nests. At hatching, within‐nest variation in hatching order emerged as the primary predictor of sex ratio, with later‐hatched offspring more likely to be female in larger broods when one or more eggs failed to hatch; such broods were primarily produced in years of abundant food. No evidence of direct sex‐dependent mortality among nestlings was observed between hatching and fledging; instead, sex‐independent mortality of nestlings increased for offspring that hatched later in a brood and during years of low *Apodemus* and *Microtus* prey abundance. At fledging, the primary predictor of offspring sex ratio was year‐to‐year variation in food abundance, with more male fledglings produced in years of abundant food and larger broods, but only in nests where one or more nestlings had died. We found no compelling evidence for within‐season variation in offspring sex ratio between early‐ and late‐season nests. Our findings suggest that offspring sex ratios in raptors are shaped by a complex interplay of maternal adjustments and environmental influences, particularly food abundance, which drives changes in brood size. These findings emphasize the need for future research to conduct a more comprehensive examination into offspring sex adjustments, particularly focusing on alterations in sex ratio during multiple nesting stages and their association with variation in offspring mortality and environmental conditions.

## Graphical abstract

Investigating within‐season and among‐year variations in offspring sex ratios and how these variations change during multiple stages of the nesting cycle, from hatching to fledging, was the main objective of the study. To achieve this, we used the Boreal Owl as a model species. We found that within‐nest variation in hatching order and the occurrence of egg loss within a brood were the primary predictors of sex ratio at hatching, while among‐year variation in food abundance and the presence of nestling mortality within a brood were the primary predictors of sex ratio at fledging. Our findings highlight promising directions for future research into the mechanisms of sex‐ratio manipulation in animals and emphasize the need for a more comprehensive examination of offspring sex adjustments.

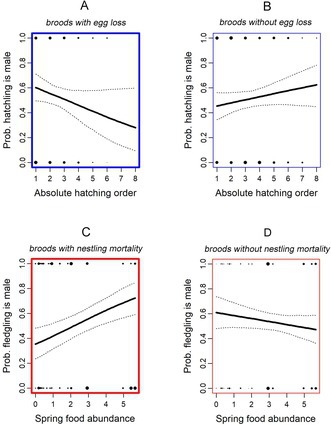

## 1 Introduction

Offspring sex ratios should vary adaptively under different environmental conditions when the costs and benefits of rearing sons and daughters differ. The theoretical basis for this idea was introduced by Fisher (1930), who suggested that sex ratios are shaped by frequency‐dependent selection, leading to equal parental investment in each sex by the end of parental care. This idea was further elaborated on by Trivers and Willard (1973), who noted that changes in environmental conditions can alter the relative costs and benefits of raising each sex, resulting in deviations from equal offspring sex ratios. These concepts have been frequently tested, especially in raptors, where sexual size dimorphism (Newton 1979) creates differing costs for rearing male and female offspring, and asynchronous hatching can facilitate sex ratio adjustments even after the primary sex ratio (at fertilization) is established. However, the selective pressures identified as driving adaptive offspring sex ratios remain inconsistent across studies, even among species with similar ecology and physiology (Table 1), leaving it unclear whether observed differences reflect within‐ or among‐year variation and whether this variation is linked to the predictability of environmental conditions. This uncertainty arises from two weaknesses in existing research: studies on variation in offspring sex ratios are either short‐term, providing a limited understanding of among‐year consistency, or focused on a small portion of the nesting cycle, providing limited information about when sex ratios were established or altered.

**TABLE 1:** Detailed information from a survey of studies of offspring sex ratios in raptors. The information documents among‐study variation in response variables, predictor variables, the completeness/incompleteness of offspring sex identification, nesting phases to which the results were interpreted, and the duration (number of years) of each study. ‘Yes’ indicates that the information within a column was included in the publication. Significant predictors are shown in bold font. The table contains studies found using Google Scholar with the keywords “offspring sex ratio, sex‐biased nestling mortality, sex allocation, food conditions, seasonal variation, birds of prey, raptors, and owls”, and only those that included at least one targeted predictor variable (hatching date, food abundance, year, and hatching order). However, additional studies known to us, but not identified by searching with Google Scholar, are also included.

Authors	Species	Response variable		Predictor variable						Completeness of offspring sex identification			Nesting phase related to result interpretation					Study duration
		Offspring sex ratio	Sex‐related offspring mortality	Hatching (laying) date	Food abundance	Year	Hatching order	Presence/absence of egg loss and nestling mortality	Other predictor(s)	Complete	Incomplete	Unclear	Laying	Hatching	Fledgling	Difference among nesting stages	Unclear	Number of years
Byholm, Brommer et al. (2002)	*Accipiter gentilis*	Yes		**Yes**		Yes			Yes		Yes						Yes	10
Rutz (2012)	*Accipiter gentilis*	Yes		Yes	Yes	**Yes**	Yes		Yes	Yes				Yes				5
Byholm, Brommer et al. (2002)	*Accipiter gentilis*	Yes			**Yes**				Yes	Yes					Yes			10
Kouba et al. (2020)	*Aegolius funereus*	Yes		Yes	Yes		Yes		Yes			Yes					Yes	7
Schwerdtfeger and Wink (2014)	*Aegolius funereus*	Yes		Yes	Yes^b^		Yes		Yes			Yes					Yes	11
Hipkiss and Hörnfeldt (2004)	*Aegolius funereus*	Yes		Yes	**Yes** ^c^	**Yes**			Yes	Yes				Yes				3
Hipkiss et al. (2002)	*Aegolius funereus*		Yes	Yes	**Yes** ^c,^ d	**Yes** ^c,^ d			Yes	Yes				Yes				2
Hörnfeldt et al. (2000)	*Aegolius funereus*	Yes	Yes^a^	Yes^SR^	Yes^SR^		Yes		Yes	Yes	Yes			Yes	Yes	Yes		1
Väli (2012)	*Aquila pomarina*	Yes			**Yes**	Yes			Yes		Yes				Yes			18
Tschumi et al. (2019)	*Athene noctua*	Yes	Yes				**Yes** ^**SR**^ Yes^M^			Yes	Yes			Yes	Yes	Yes		5
Humbel (2014)	*Athene noctua*	Yes	Yes	**Yes** ^**SR**^ Yes^M^					Yes		Yes			Yes				5
Mora et al. (2010)	*Bubo bubo*	Yes		**Yes**	Yes	Yes	Yes		Yes			Yes					Yes	5
Penteriani et al. (2010)	*Bubo bubo*	Yes					**Yes**		Yes			Yes		Yes				5
Chakarov et al. (2015)	*Buteo buteo*	Yes		Yes	**Yes** ^e^				Yes	Yes					Yes			11
Zijlstra et al. (1992)	*Circus aeruginosus*	Yes		**Yes**			Yes		Yes	Yes	Yes						Yes	11
Millon and Bretagnolle (2005)	*Circus pygargus*	Yes		Yes	**Yes**				Yes	Yes	Yes				Yes			16;8
Arroyo (2002a)	*Circus pygargus*		Yes	**Yes**			Yes		Yes		Yes			Yes				8
Arroyo (2002b)	*Circus pygargus*	Yes		Yes	**Yes**		Yes			Yes					Yes			7
Ristow and Wink (2004)	*Falco eleonorae*	Yes		**Yes**								Yes					Yes	5
Olsen and Cockburn (1991)	*Falco peregrinus*	Yes		**Yes**					Yes			Yes					Yes	14
Griggio et al. (2002)	*Falco sparverius*	Yes		**Yes**		Yes				Yes					Yes			29
Smallwood and Smallwood (1998)	*Falco sparverius*	Yes	Yes^a^	**Yes** ^**SR**^							Yes			Yes	Yes	Yes		4
Wiebe and Bortolotti (1992)	*Falco sparverius*	Yes	Yes^a^	Yes^SR^	**Yes** ^**SR,c**^	**Yes** ^**SR**^			Yes	Yes	Yes		Yes	Yes	Yes	Yes		3
Wu et al. (2010)	*Falco tinnunculus*	Yes		**Yes**			Yes		Yes	Yes	Yes		Yes	Yes	Yes	Yes		2
Rejt et al. (2005)	*Falco tinnunculus*	Yes		**Yes**					Yes	Yes	Yes		Yes	Yes	Yes	Yes		2
Laaksonen et al. (2004)	*Falco tinnunculus*	Yes		Yes	Yes^4^			**Yes** ^f^	Yes	Yes	Yes						Yes	8
Korpimäki et al. (2000)	*Falco tinnunculus*	Yes		**Yes**	**Yes** ^c^	**Yes**			Yes	Yes	Yes			Yes				3
Dijkstra et al. (1990)	*Falco tinnunculus*	Yes		**Yes**			**Yes**		Yes	Yes	Yes						Yes	7
Gómez‐López et al. (2023)	*Gyps fulvus*	Yes		Yes		Yes^c^			Yes		Yes			Yes				28
Blanco et al. (2002)	*Otus Scops*	Yes		Yes		Yes	**Yes**		Yes		Yes		Yes	Yes		Yes		4
Schreven et al. (2022)	*Pernis apivorus*	Yes		**Yes**	Yes		Yes		Yes	Yes	Yes			Yes				18
Sasvári and Nishiumi (2005)	*Stric aluco*	Yes	Yes			**Yes**	**Yes** ^**SR,M**^		Yes	Yes				Yes	Yes	Yes		11
Tooth et al. (2024)	*Stric aluco*	Yes		**Yes**					Yes	Yes			Yes		Yes			8
Appleby et al. (1997)	*Strix aluco*	Yes			**Yes**				Yes	Yes				Yes	Yes	Yes		2
Kekkonen et al. (2008)	*Strix aluco*	Yes		Yes	Yes^b^	Yes				Yes	Yes						Yes	5
Brommer et al. (2003)	*Strix uralensis*	Yes	Yes^a^	Yes^SR^					Yes	Yes	Yes		Yes	Yes	Yes	Yes		3

Abbreviations: M: sex‐related offspring mortality (response variable), SR: sex ratio (response variable).

^a^ Offspring mortality (response variable) was evaluated based on the offspring sex ratio at different nesting stages.

^b^ The number of prey items stored in owl nests was used as an index of food abundance.

^c^ The year was used as a predictor variable to analyze the variability in offspring sex ratios among years, while observed findings related to among‐year variations were interpreted in the context of food abundance.

^d^ A statistically significant interaction between year and supplementary feeding.

^e^ A statistically significant interaction between food abundance and plumage morph.

^f^ Authors included the predictor “completeness” to indicate whether the whole clutch was sexed or the sex of one egg was missing.

Selection for sex ratios of offspring may arise from environmental conditions that vary from the beginning to the end of the breeding season, as well as from year to year. Within‐season changes in conditions that occur every year lead to within‐season variation in diverse aspects of the breeding biology of birds, from clutch size (Klomp 1970) to the probability of survival of independent offspring (Hochachka 1990). Regular seasonal changes, associated with nest initiation dates, may also contribute to within‐season variation in the sex ratio of offspring, with selection for either male or female offspring at the beginning of breeding seasons (Daan et al. 1996; Cordero et al. 2001; Krebs et al. 2002; Andersson et al. 2003; Mora et al. 2010). In contrast, inconsistent and unpredictable changes in conditions, both within a season and across years, have driven the evolution of diverse reproductive adaptations in raptors, particularly those that primarily feed on small mammals whose population sizes fluctuate widely (Lehikoinen et al. 2011; Solonen et al. 2015). These adaptations include annual variation in offspring sex ratios, with conventional wisdom suggesting a preference for the smaller sex (males) during years of food scarcity (Wiebe and Bortolotti 1992; Korpimäki et al. 2000; Arroyo 2002a). However, this expected pattern is not consistently observed across studies (Kekkonen et al. 2008; Mora et al. 2010; Schwerdtfeger and Wink 2014), with some studies finding the opposite pattern of the smaller sex being favored in years of high abundance of food (Byholm, Ranta et al. 2002; Hipkiss and Hörnfeldt 2004; Millon and Bretagnolle 2005). Overall, there is substantial inconsistency in both the presence and direction of year‐to‐year and within‐season variation in offspring sex ratios among raptors, even among species with similar life histories (Table 1).

Adjustments to the sex ratio of offspring can occur multiple times and in different directions in response to different cues throughout the entire nesting cycle. This process can start during the production of the primary sex ratio at ovulation (Badyaev et al. 2006, 2008; Goerlich‐Jansson et al. 2013) and continue during incubation due to elevated embryonic mortality of one sex (Love et al. 2005). More easily studied and more frequently reported is sex‐related variation in offspring mortality after hatching, which produces the secondary sex ratio. For example, adults can actively control nestling mortality through parents' control of provisioning (Stamps et al. 1985) or through direct sibling competition, where adults passively provide more food to the nestling exhibiting more prominent begging behavior (Grodzinski and Lotem 2007). In species with asynchronous hatching and an increased likelihood of the mortality of later‐hatched and smaller nestlings, an additional opportunity exists to adjust the sex ratio of fledglings by altering the sex ratio through the laying sequence so that later‐hatching chicks are more likely of the sex to experience sex‐biased mortality in competition with older siblings (Bortolotti 1986; Bradbury and Griffiths 1999; Sasvári and Nishiumi 2005). The patterns of adjustment in sex ratio may also be more complex depending on clutch and brood sizes. For example, the mortality of last‐hatched nestlings may be more likely in larger broods (Tschumi et al. 2019), where substantial size discrepancies exist between oldest and youngest nestlings. Food availability will affect clutch and brood sizes (Newton 1979), and hence the magnitudes of size discrepancies. Despite these insights, the associations between the mechanisms that animals use to adjust sex ratios and the selective pressures driving these adjustments remain poorly understood.

We suggest that the timing and mechanisms for adjusting sex ratios may depend on the predictability of selection on sex ratio. Predictable changes in environmental conditions within the breeding season that are consistent across years (e.g., those mediated by photoperiod or relative nest initiation date) would lead to adjustments in offspring sex ratios early in the nesting cycle, preventing parents from investing in rearing offspring of a sex that would die with higher frequency before the end of parental care. In contrast, unpredictable changes in environmental conditions (such as food abundance) may lead to adjustments in sex ratios later in the nesting cycle through sex‐biased mortality, allowing parents to maximize the survival prospects of the more beneficial sex. Without following patterns of variation in sex ratios from as close to the time of ovulation as possible, the relative contributions of different mechanisms leading to the final sex ratios of offspring cannot be identified. Therefore, we believe the following three hypotheses should be examined together:

1. In populations with substantial variation in nest initiation dates, within‐season variation in offspring sex ratio will exist across nests started at different dates during the breeding season, and the qualitative pattern of this variation will be consistent across years.
2. In populations experiencing large year‐to‐year and within‐season fluctuations in food abundance, a corresponding variation in offspring sex ratio will be prominent in sexually size‐dimorphic species.
3. Variation in offspring sex ratio associated with year‐to‐year consistent selective pressure (nest initiation dates) will be established early in the nesting cycle (i.e., before eggs hatch). Modifications in offspring sex ratio associated with unpredictable variation in food abundance will occur later in the nesting cycle (i.e., after eggs hatch) due to the mortality of nestlings; given asynchronous hatching of eggs in raptor nests, sex‐biased mortality should be more prominent for later‐hatched nestlings from larger broods.

To test the above hypotheses, we will use 18 years of data on 542 Boreal Owl ( *Aegolius funereus* ) offspring from 140 nests in Central Europe. The Boreal Owl is an appropriate model species with which to examine multiple independent alterations of offspring sex ratios throughout a nesting cycle and to explore within‐season and year‐to‐year variation in these patterns for several reasons. First, Boreal Owl parents and their nestlings exhibit sexual‐size dimorphism with larger females than males (Korpimäki 1986; Hipkiss 2002; Zárybnická, Riegert, Brejšková 2015), which may lead to higher mortality rates among male offspring due to competition with their larger female siblings (Hipkiss et al. 2002). Second, asynchronous hatching and the resulting size hierarchy among nestlings could potentially lead to the mortality of the youngest offspring of one sex when food is limited. Third, their breeding period is spread over several months, with egg‐laying starting between March and June and decreasing clutch size and reproductive output through this time (Zárybnická et al. 2015). The wide range of laying dates could lead to differences in sex ratios between the early and late nests. Fourth, the reproductive life history of the Boreal Owl strongly depends on the abundance of *Microtus* voles and *Apodemus* mice (Korpimäki 1992; Eldegard and Sonerud 2009; Zárybnická and Vojar 2013; Zárybnická et al. 2015), which varies substantially among years (Berryman 2002; Zárybnická et al. 2013). This high variability in food availability may result in differences in offspring sex ratios between years with low and high food abundance, typically associated with smaller and larger broods. Some previous research of Boreal Owls has identified one of the patterns for which we are testing in our paper, with Hipkiss et al. (2002) and Hipkiss and Hörnfeldt (2004) having found associations between inter‐annual variation in food availability and variation in sex‐dependent mortality. However, the lack of statistical detection in other studies of food‐related variation in offspring sex ratios (Schwerdtfeger and Wink 2014; Kouba et al. 2020), or within‐season variation in offspring sex ratios (Hipkiss and Hörnfeldt 2004; Schwerdtfeger and Wink 2014; Kouba et al. 2020) is difficult to interpret as being biologically real or merely an artifact of methodological constraints, such as short‐term or stage‐specific studies, or the interplay of biological predictors.

## 2 Material and Methods

### 2.1 Study Site

The study was conducted in northwest Czechia (50.7° N, 13.6° E) on the Ore Mountain plateau (700–920 m a. s. l.) from 2006 to 2024, with no data available for 2013 due to a complete reproductive failure caused by food scarcity. The study area consisted of small patches (usually 0.5–2.0 ha) of mature Norway spruce *Picea abies* , old solitary European beech *Fagus sylvatica* trees, and large areas of young coniferous trees (mainly the non‐native blue spruce *Picea pungens* , European larch *Larix decidua* , and native Norway spruce) and deciduous trees (mainly birches *Betula* spp., European mountain ash *Sorbus aucuparia* , and European beech). The habitat composition resulted from extreme SO_2_ and NO_x_ pollution emitted in the 1970s–80s from factories in the Ore Mountain foothills (Šrámek 1998). In this study site, Boreal Owls breed mainly in nest boxes (> 90% of breeding pairs) due to the limited availability of large trees with natural cavities (Zárybnická, Riegert, Šťastný 2015). In 2006–2024, the number of nest boxes available for Boreal Owls varied between 116 and 246 (mean ± SE; 194.5 ± 10.4 per year), well in excess of the number of nests each year.

### 2.2 Owl Reproductive Data

We checked nest boxes every year from the onset (late March) to the end (late August) of the breeding season to find active nests and obtain information on hatching date, clutch size, brood size (the number of hatchlings), and the number of fledglings. Once we discovered an owl nest, it was checked weekly to determine hatching date, hatching order, egg loss, nestling mortality, and to ring (i.e., band) nestlings. From 2006 to 2024, we monitored 162 Boreal Owl broods (i.e., nests with hatched offspring) and collected blood samples from 542 nestlings in 140 of these broods. During the nestling period, one of these broods was depredated by Pine Marten *Martes martes* , and four broods were abandoned. Clutch size varied from two to eight eggs (mean ± SD, 4.9 ± 1.3, *n* = 140 clutches) and brood size from one to eight fledglings (3.5 ± 1.9, *n* = 135 broods). Egg loss, referring to the number of unhatched eggs without a known cause of failure (i.e., not abandoned or depredated), was 0.6 ± 0.8 eggs per clutch (*n* = 140 clutches) and nestling mortality was 0.8 ± 1.1 nestlings per brood (*n* = 135 broods). Depending on the statistical model, we analyzed data from 101 to 135 broods, consisting of 424–523 nestlings (for details, see Statistical analyses, Table 2).

**TABLE 2:** A summary of sample sizes used in analyses, separated by individual models (Models 1–3) and for all data pooled. The probability of the sex ratio at hatching and fledging is examined in Models 1 and 3, respectively, and the probability of offspring mortality is examined in Model 2.

Characteristic	All data	Model 1	Model 2	Model 3
No. offspring (male/female)	542	424 (221/203)	523 (274/249)	466 (247/219)
No. broods	140	101	135	135
No. years	18	17	18	18
Hatching date: mean (SD)	May 23 (27.4 days)			
Spring food index: min; max	0; 5.6			
Spring‐autumn food index: min; max	−3.7; 5.5			
No. broods without egg loss (No. hatchlings in these broods)		50 (258)		
No. broods with egg loss (No. hatchlings)		51 (166)		
No. broods with complete sexing (No. nestlings)			99 (413)	
No. broods with incomplete sexing (No. nestlings)			36 (110)	
No. broods without nestling mortality (No. nestlings)				73 (304)
No broods with nestling mortality (No. nestlings)				62 (162)
Clutch size: mean per brood (SD)		4.9 (1.4)		
Brood size: mean per brood (SD)		4.2 (1.7)	4.3 (1.6)	
No. fledglings: mean per brood (SD)			3.5 (1.9)	3.5 (1.9)
No. eggs failed to hatch (all broods): mean per brood (SD)		0.6 (0.8)		
No. eggs failed to hatch (only broods with egg loss): mean per brood (SD)		1.4 (0.6)		
No. unsexed nestlings (only broods with incomplete sexing): mean per brood (SD)			1.5 (0.9)	
No. died nestlings (all broods): mean per brood (SD)				0.8 (1.1)
No. died nestlings (only broods with nestling mortality): mean per brood (SD)				1.8 (1.0)

*Note:* “No.” means the number.

We trapped 134 female and 122 male parents from 140 broods, ringing any adults previously unringed. There were 112 unique female and 86 unique male adults trapped, of which 13 females and 22 males nested multiple times, either within a season or over several years. Of these repeated records of nesting, there were only two cases in which the same female and male re‐paired in two different years and one case in which the same female and male re‐paired in three different years. No identical female and male re‐paired in the same year. We failed to trap six female and 19 male parents in 23 nests. Based on examining information about the nests with untrapped adults (e.g., year, time, nest location, and distance to nearest nests), we dismissed the potential that these birds had nested in our study area multiple times. Therefore, we added a unique ID in our data for each of these adults in our statistical analyses.

### 2.3 Estimating the Hatching Date and Order

Throughout our study, the same person (MZ) used the same process to determine the hatching date of the first hatchling in a nest, from which hatching dates of subsequent nestlings within a nest were calculated. The information used to calculate the hatching date of the first nestling came from checking nests, which usually was carried out at one‐week intervals during the period when we expected hatching to occur or nestlings to be present. Occasionally, the intervals between nest checks were shorter or longer than 1 week, but longer intervals did not prevent us from estimating the date of hatching of the first eggs. In some cases, we recorded the exact date of hatching of the first nestling. For most nests, we needed to calculate the first‐hatch date based on other information. For these estimates, we compared the measured wing lengths and body masses of first‐hatched nestlings to growth curves developed by Drdáková (2005) and Zárybnická, Riegert, Brejšková (2015). In the first step of this process, we estimated the hatching date using the sex‐independent growth curve of wing length because of the low variability in wing‐length measurements and the very similar rate at which wing length grows between sexes. Afterward, we verified that these estimates of hatching dates were consistent with the sex‐dependent growth curves of body mass; the growth of nestling mass is more variable between sexes than the growth of wing length, and simultaneously, the growth of nestling mass, relative to the growth of wing length, is much more variable even within a single sex. Therefore, the estimates of hatching dates based on mass growth were only used to check measurement errors in wing length or highly abnormal growth patterns. Finally, we corroborated the estimates of the first‐hatch date based on the growth curves using additional information, such as the timing of laying of the first egg, the duration of the incubation period, and the stage of plumage development that allowed us to recognize different‐aged nestlings by their gradual replacement of white protoptile (first) and gray mesoptile (second) down plumage, and the growth of brown primary flight feathers (Mikkola 2014).

We derived the hatching date of the second and subsequent hatchlings in each brood from the hatching date of the first hatchling based on a typical interval between the hatching of eggs that we determined from nest‐box cameras installed in 19 nests in 2014–2017 (Zárybnická et al. 2016). For these nests, the hatching interval of 70 hatchlings was 1.4 ± 0.9 days (range 0–3 days, median = 1 day). Therefore, we used an interval of 1 day to estimate the hatching dates of all nestlings after the first in a nest. To determine the hatching order (sequence), we used comparisons of wing length, body mass, and plumage development of nestlings in each brood; there was a clear and linear hierarchy in size and plumage development that allowed us to rank nestlings from oldest to youngest.

### 2.4 Egg Loss and Nestling Mortality

Studies examining variation in offspring sex ratio in raptors regularly encounter difficulties in obtaining complete sex‐related data before offspring mortality occurs (e.g., Wiebe and Bortolotti 1992; Laaksonen et al. 2004; see Table 1 for more examples). Some authors (Fiala 1980) have argued that subsets of data containing only completed sex ratios at one age cannot reliably establish sex ratios at an earlier age across all nests, as offspring mortality may be sex‐dependent and thus sex ratios may differ between clutches with and without egg loss. We test for these possibilities in our analyses using two approaches. First, when fitting models for offspring sex ratios (for details, see Statistical analyses), we examined whether our estimates of sex ratio were sensitive to the inclusion of information from clutches in which some egg(s) failed to hatch compared to clutches in which all eggs hatched (referred to as the binomial predictor “eLoss”). Alternatively, we tested whether sex ratio estimates differed in broods in which some nestlings died between hatching and fledging and those in which all nestlings survived to fledge (“nMort”). Second, in the models of nestling mortality (for details, see Statistical analyses), we included an additional binomial predictor (“completeSexing”) to control for potential differences between broods in which the sexes of some nestlings were unknown because they died before we took blood samples and broods in which the sexes of all nestlings were determined.

### 2.5 Molecular Sex‐Determination

For the molecular determination of offspring sex, we collected blood samples from live nestlings' brachial veins. These blood samples were typically collected at 2 weeks of age for each nestling (median = 14 days); the nestling period is typically 28–35 days long (Drdáková 2003). In clutches where some eggs failed to hatch, we did not determine the sex of unhatched nestlings. In broods experiencing any nestling mortality, we determined the sexes of all living nestlings, including those that later died. Some younger nestlings died soon after hatching and before our nest visits for blood samples; thus, we could not sex 50 hatchlings in 33 broods (9.5% of all hatchlings).

We determined the sexes of nestlings using PCR screening based on the amplification of an intron from the sex chromosome‐linked CHD1 gene, which differs in size on the Z and W chromosomes (Fridolfsson and Ellegren 1999). We extracted genomic DNA from blood samples (stored in 96%–100% ethanol and a −20°C freezer) by alkaline lysis followed by a neutralization step (Truett et al. 2000). The supernatant was directly used as a template for PCR. We prepared the PCR premix according to the Taq DNA Polymerase (New England Biolabs) manufacturer's guidelines (samples from 2006 to 2014) and according to the LA Hot Start Master Mix (Top‐Bio) manufacturer's protocol (samples from 2015 to 2024), using the primers 2550F (5'‐GTTACTGATTCGTCTACGAGA‐3′) and 2718R (5'‐ATTGAAATGATCCAGTGCTTG‐3′) (Fridolfsson and Ellegren 1999). The PCR was conducted under the following conditions: initial denaturation for 5 min at 95°C, followed by 31 cycles of denaturation at 95°C for 30 s, annealing at 60°C for 40 s, an extension at 72°C for 1 min 10 s, and a final extension at 72°C for 5 min. The amplified fragments were separated by gel electrophoresis (1.5% agarose gel stained with ethidium bromide, 100 V, 45 min) and visualized using UV illumination. Males showed a single Z‐band (700 bp) and females displayed Z‐ (700 bp) and W‐ (1200 bp) bands (Fridolfsson and Ellegren 1999). Each PCR run included two positive controls (DNA template of an adult female and an adult male) and a negative control.

### 2.6 Small‐Mammal Abundance

**FIGURE 1:**
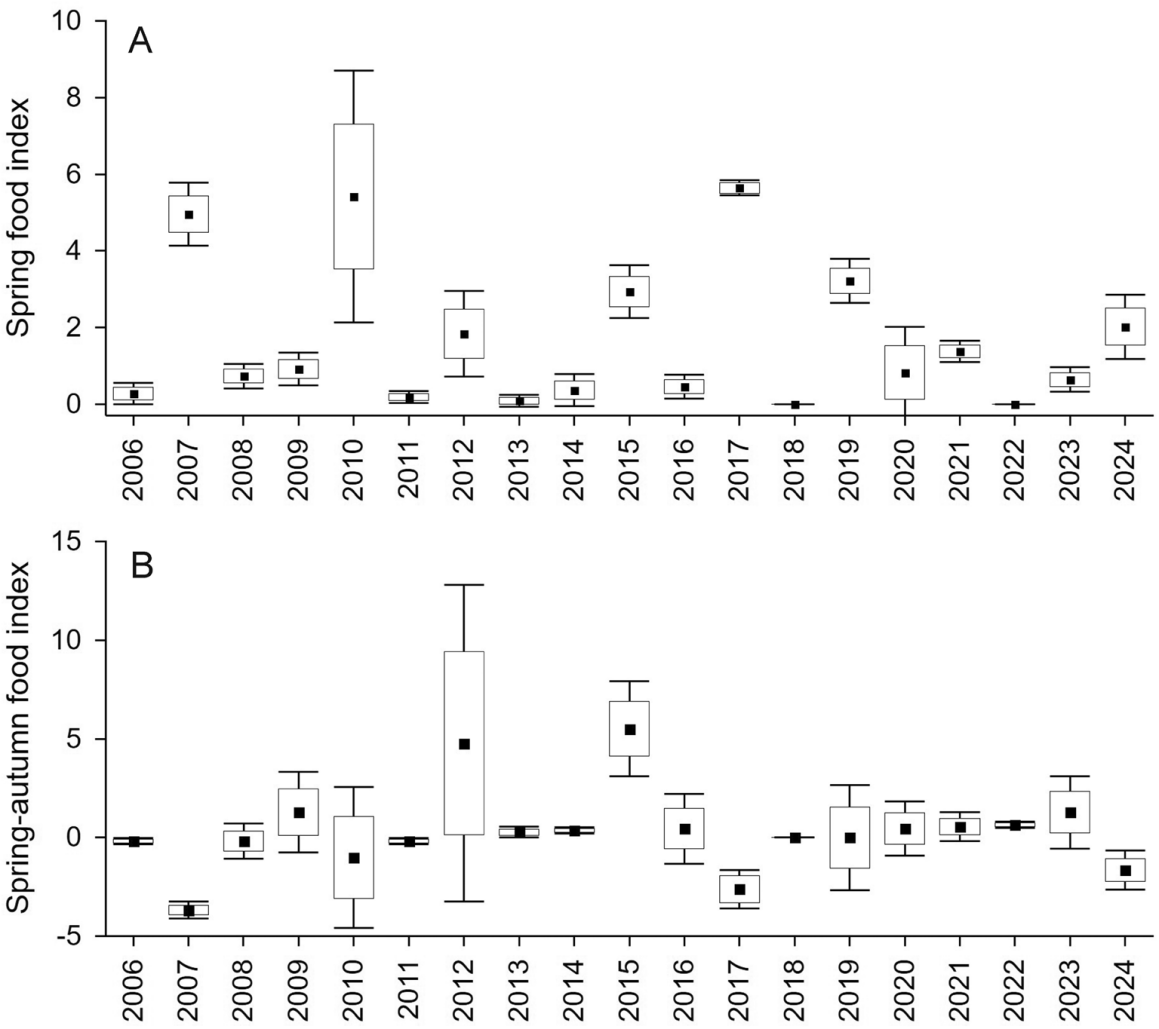
Abundance and seasonal changes in abundance of small mammals during the period of 2006–2024 in the study area. (A) The spring food index was calculated for each year as the mean number of pooled *Apodemus* mice and *Microtus* voles captured per 100 trap nights across three trapping sites during spring (early June) trapping events. This index was used as the predictor *Spring food abundance* in Models 1–3. (B) The spring‐autumn food index was calculated for each year as the seasonal change in the number of pooled *Apodemus* mice and *Microtus* voles captured per 100 trap nights across three trapping sites during spring (early June) and autumn (early October) trapping events. Negative values reflect a decrease in food abundance from spring to autumn, and positive values reflect an increase in food abundance from spring to autumn. This index was used as the predictor *Spring‐autumn food abundance* in Models 1–3. The points, boxes, and whiskers indicate mean, SE, and SD.

We used snap‐trapping during the breeding period to create indices of abundance of *Apodemus* mice and *Microtus* voles in representative sites within the study area: our past work documents that these trapping data provide an appropriate index of food availability for Boreal Owls to examine variations in their reproductive characteristics (Zárybnická et al. 2009; Zárybnická et al. 2013; Zárybnická, Riegert, Brejšková 2015; Zárybnická, Riegert, Kouba 2015; Zárybnická et al. 2015; Kouba et al. 2017). Snap‐trapping was conducted each spring at the beginning of June and each autumn at the beginning of October. We set up snap traps at three 1‐ha blocks (11 × 11 traps, 10‐m between traps) and operated the traps for three nights, checking them every morning (note that only two, instead of the typical three, trapping blocks were operated in 2017). To eliminate the negative effect of trapping individuals within the same 1‐ha squares during spring and autumn of the same year, we placed the autumn trapping squares in areas neighboring the spring trapping squares. We identified all trapped individuals to the species level and then grouped them into five categories (*Apodemus* mice, *Microtus* voles, *Sorex* shrews, *Clethrionomys* voles, and others). From 2006 to 2024, we trapped a total of 244 *Apodemus* mice and 83 *Microtus* voles during spring trapping events and 201 *Apodemus* mice and 202 *Microtus* voles during autumn trapping events, with numbers fluctuating substantially among years (Figure 1). We calculated two abundance indices for each year. First, a nesting‐season food abundance index—referred to as “spring food index (abundance)” and “food abundance”—was defined as the number of trapped *Apodemus* and *Microtus* per 100 trap nights in each trapping block and event during spring trapping. Second, a food abundance index that illustrates seasonal changes in food conditions from spring to autumn—referred to as “spring‐autumn food index (abundance)” and “seasonal changes in food abundance”—that we defined as the difference between the number of trapped *Apodemus* and *Microtus* per 100 trap nights in each trapping block and event during spring trapping and autumn trapping. Values for the second index ranged from negative to positive, with negative values reflecting a decrease in food abundance from spring to autumn and positive values reflecting an increase in food abundance from spring to autumn. We included both the spring food index, which varied yearly from 0 to 5.6 (Figure 1A), and the spring‐autumn index, which ranged from −3.7 to 5.5 among years (Figure 1B), in the statistical analyses.

### 2.7 Statistical Analyses

To examine our hypotheses, we used the data on individual offspring sex and nestling mortality summarized in Table 2. Following the convention of previous studies (e.g., Appleby et al. 1997; Millon and Bretagnolle 2005; Schreven et al. 2022), we used the term “sex ratio” when we referred to the probability of an offspring being male or female. We used R (Version 4.4.2; R Development Core Team 2013) to fit three generalized linear mixed models (GLMMs) with binomial error distributions, using the *glmer* function from the *lme4* package (Bates et al. 2012). Using the first model, we explored the probabilities of individual offspring being male at hatching (secondary sex ratio), including an examination of whether the sex ratio of hatchlings was altered due to embryo loss during incubation. With the second model, we estimated the probability of an individual offspring dying between hatching and fledging. Finally, using the third model, we examined the probabilities of individual offspring being male at fledging, including an examination of whether the sex ratio of fledglings was altered due to nestling mortality. Together, these three models allowed us to assess the extent to which the sex ratio of offspring changed from hatching to fledging.

By treating individual offspring instead of nests as the units of analysis, we were able to test the effect of hatching order on offspring sex ratio and the effect of the sex of later‐hatched nestlings in individual nests on their mortality. However, we needed to account for potential statistical non‐independence of offspring of the same parent. We addressed this issue by including the IDs of the female and male parents as random intercept effects. Given that a small proportion of male and female parents (15.7% of adults) nested multiple times, the random effects of ID parents simultaneously accounted for non‐independence of offspring in each individual nest (84.3% of adults have data from only one nest) as well as across the multiple nests of some adults. We also included the calendar year as a random effect to control for sources of among‐year variability not accounted for by any of the fixed effects. Since no pair nested together more than once in the same year, using male ID, female ID, and year as random effects also accounted for non‐independence of offspring within individual nests. In our preliminary analyses, we explored the use of nests, rather than parents' identities, as a random effect to account for non‐independence of siblings in the same nest, and we found that the residual variance attributed to a nest's random effect was negligible compared to the variance ascribed to individual parents. Nevertheless, the fixed effects of the models with nests included as a random effect were qualitatively identical to models with the IDs of individual parents and year as random effects.

We used the sex of offspring (coded: 0 = male, 1 = female) as a binomial response variable to calculate the probabilities of each individual offspring being male at hatching (first model) and at fledging (third model). In these two models, we included the following continuous fixed‐effects: hatching date (recorded for each offspring as the number of days since January 1) and the two indices of food abundance—the spring food index to quantify the abundance of pooled *Apodemus* and *Microtus* individuals during the respective breeding (spring) seasons and the spring‐autumn food index to assess whether within‐season changes in the abundance of pooled *Apodemus* and *Microtus* affected nesting. We also included offspring hatching order as ordinal predictor, treating this variable in two ways. Primarily, we treated the hatching order as a continuous (first to hatch = 1, second to hatch = 2, etc. in each brood) predictor based on the assumption that the combination of hatching asynchrony and brood size, together resulting in a hierarchy in nestling size, led to monotonic changes (i.e., either continually increasing or decreasing) in responses to hatching order. We referred to this predictor as “absolute hatching order,” indicating that its values were associated with brood size and that the last‐hatched nestling in a small brood experiences a different nest environment compared to a last‐hatched nestling in a large brood (i.e., there is a smaller disparity in age and size between first‐ and last‐hatched nestling and fewer competitors for parental care in smaller broods than larger broods). In a second set of parallel analyses, we treated hatching order as a continuous value between 0 and 1, where 0 represents the first‐hatched nestling and 1 represents the last‐hatched nestling (the specific hatching order was divided by brood size), to examine whether the sex ratio monotonically changed in response to hatching asynchrony independent of brood size. We referred to this predictor as “relative hatching order,” indicating that the last‐hatched nestling in a small brood was treated as if it experienced a nest environment similar to that of the last‐hatched nestling in a large brood. Absolute and relative hatching orders were highly correlated (*r* > 0.7), which is why we created two parallel sets of analyses with which to examine each of our three response variables (three models), one set including absolute hatching order and the other including relative hatching order.

When modeling the sex ratio at hatching, we also included information on whether any egg loss (“eLoss”) occurred during incubation. This binomial predictor variable allowed us to test whether estimated sex ratios at hatching differed depending on whether any egg(s) in a clutch failed to hatch or not (1 = no egg loss, 0 = at least one egg did not hatch). Clutches with no egg loss were the reference category, that is, the parameter estimate for the effect of “eLoss” described the deviation in the probability of an offspring being male for clutches with any egg loss relative to clutches in which no egg(s) were lost prior to hatching. This first model compared the 50 clutches without egg loss to 51 clutches with egg loss (where an average of 1.4 eggs per clutch failed to hatch); females laid a total of 497 eggs and produced 424 hatchlings in these broods, respectively (Table 2).

For modeling the sex ratio at fledging (third model), we included information on whether any nestling mortality (“nMort”) occurred between hatching and fledging. This binary predictor variable allowed us to test whether there was an effect of the existence of any nestling mortality in individual broods (1 = no nestling mortality occurred, 0 = at least one nestling did not fledge) on the sex ratio at fledging. Broods with no nestling mortality were the reference category, that is, the parameter estimate for the effect of “nMort” described the deviation in the probability of an offspring being male for broods with any nestling mortality relative to broods in which no nestling died prior to fledging. The model included data from 73 and 62 broods without and with nestling mortality: a total of 304 and 162 offspring hatched in these broods, respectively (Table 2). In both the first and third models, we used the IDs of male and female parents and year as random effects.

The second model calculated sex‐specific differences in the probability that a nestling would die between hatching and fledging, in which the response variable was nestling mortality (coded: 1 = the nestling died, 0 = the nestling fledged). The fixed‐effect predictor variable of interest was the sex of a nestling (a binary variable, 0 = male, 1 = female); we used the male sex as a reference category. Continuous fixed effects were hatching date, the spring food index (quantifying the abundance of pooled *Apodemus* and *Microtus* individuals during the respective spring), the spring—autumn food index (quantifying the seasonal variation in the abundance of pooled *Apodemus* and *Microtus* in the respective year), and both absolute and relative hatching orders (in parallel models). The analysis used data from 523 hatchlings from 135 broods, for which the sex was determined (Table 2). However, 36 of these broods contained at least one hatchling that was unsexed due to its mortality before blood sampling (i.e., broods with incomplete sexing); in these broods, a total of 50 hatchlings were not sexed (mean ± SD, 1.5 ± 0.9 hatchlings per brood; 9.4% of all hatchlings). To test whether incomplete sexing was the result of early sex‐biased mortality, we added a binary variable “completeSexing” (1 = all hatchlings sexed, 0 = at least one or more nestlings were not sexed) for each brood in the model. Nests with all hatchlings sexed (*n* = 99 broods) were modeled as the reference category. Other predictors were two‐way interactions between the sex and each of the continuous predictors (hatching date, absolute or relative hatching order, spring food index, and spring‐autumn index), which test whether any of these predictors affect the sexes differently (i.e., interactions are statistically important) or not. We again used the IDs of male and female parents and year as random effects.

Prior to fitting each model, we scaled the original values of all continuous predictors to have a mean of 0 and a standard deviation of 1 within each model's data set, allowing the comparison of estimates within models. Then, we examined correlations among all scaled continual predictors. Most correlations were low (*r* ≤ 0.3), except for brood size, which exhibited high correlations with both food abundance (*r* ≥ 0.6) and hatching order (*r* ≥ 0.5). Therefore, we did not include brood size as a predictor in any model. We will interpret our results in light of these correlations. Finally, we checked multicollinearity among all fixed effects (including interactions) using variance inflation factors (VIF) with the *check_collinearity* function of the R package *performance* (Lüdecke et al. 2021): we found low multicollinearity of the predictors that were used in all models, apart from Model 2 in which the predictor *Absolute hatching order* was moderately correlated with spring food abundance.

For all of the GLMM models, we calculated the significance of regression coefficients based on estimating their confidence limits. Confidence limits were calculated by parametric bootstrapping (from 1000 parametric bootstrapped samples of the original data) because estimating *p*‐values in this way is more reliable than using the *p*‐values estimated by *glmer* (Bates et al. 2015), the R function that we used to fit the GLMMs. Prediction intervals were calculated across the range of observed values of the focal predictor variable from 1000 bootstrapped samples of the original data, refitting the model with each bootstrapped sample and recalculating the predicted values. For this, we used the function *bootMer* (with arguments *u* = *FALSE* and *type* = “*parametric*”). The 95% prediction interval around each value of the focal predictor variable was calculated as the lower (2.5%) and upper (2.5%) percentiles of the distribution of predicted values from the bootstrapped data. For these bootstrap predictions, we only estimated variation in the fixed effects (by using the *re.form = NA* argument to the *predict* function in *lme4*) because we were only interested in quantifying the uncertainty around the estimated effects of the fixed‐effect predictor variables.

### 2.8 Data Visualization

We produced figures to visualize statistically important relationships between response variables and predictors (Models 1‐3). For each variable of interest, we calculated predicted responses across the range of scaled values of the focal predictor variable, holding each non‐focal fixed‐effect variable at a constant value. While the predicted responses were calculated using scaled values, we back‐transformed predicted values to their corresponding observed (original) values for graphing. For any random effect, we identified the most typical category in the random effect (the value of the random effect coefficient for which the random effect coefficient was closest to zero). In the model describing the probability that an offspring would be male at hatching (Model 1) and fledging (Model 3), we graphed predicted effects separately for broods with and without any egg loss and nestling mortality, respectively, because the effects of these predictors were statistically significant. In the model describing the probability of nestling mortality as a function of absolute hatching order, relative hatching order, and spring food index and spring‐autumn food index (Model 2), we graphed predicted effects using data combined for male and female offspring as the effect of offspring sex was not statistically significant. For these figures, we modified the two original models (one with absolute hatching order and the other with relative hatching order) by removing the non‐significant predictor variable of offspring sex and then refitting the models (Appendix S1); parameter estimates from the displayed relationships in the modified models were qualitatively the same as those from the original models (for details, see Appendix S1). At the same time, we graphed these figures separately for broods with incomplete and complete sexing of all hatchlings because this predictor was statistically significant.

### 2.9 Ethics Statement

All methods associated with field research and animal handling were carried out under the approvals of the Ministry of the Environment of the Czech Republic (permits No. 35016/02‐OOP/8751/02, 530/758 R/08‐Abt/UL, 01220/LP/2008, 64377/FNU/2013, 4885/2017/KUUK, 48429/ENV/14–2831/630/14, MZP/2020/630/113, and 71735/ENV/16), the Ringing Centre of the National Museum in Prague (permits No. 329, 942, and 1210), and the Certificate on Prevention of Cruelty to Animals (V/2/2006/44).

## 3 Results

### 3.1 Sex Ratio at Hatching

**FIGURE 2:**
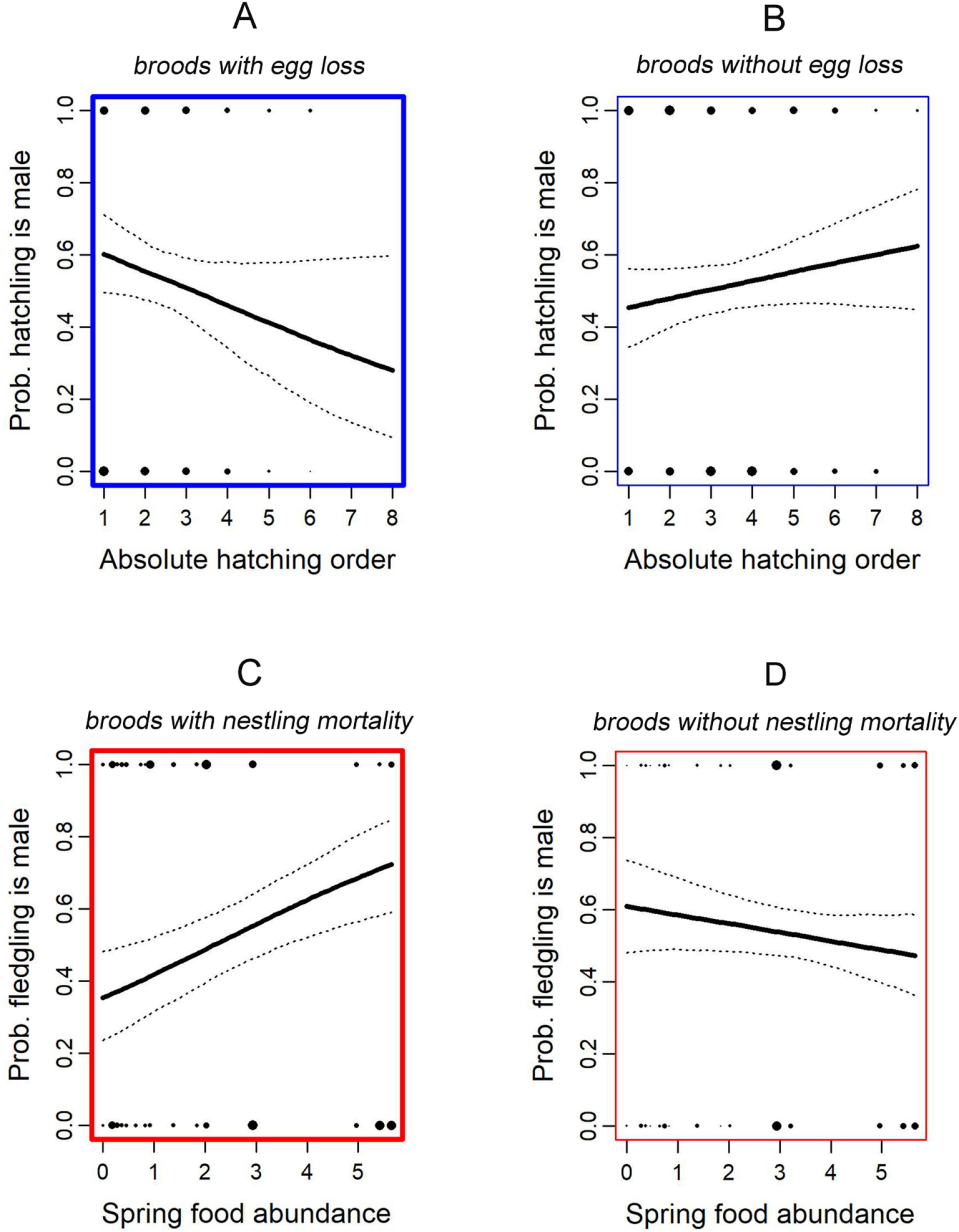
The probability of a Boreal Owl offspring being male at hatching (blue frames) and fledging (red frames). At hatching, male offspring were significantly less common among late‐hatched nestlings in larger broods in which at least one egg failed to hatch—panel A (thick blue frame, graphing Model 1 in Table 3). There was a tendency for later‐hatched nestlings to be male in larger broods without egg loss, but this effect was not statistically significant—panel B (thin blue frame). At fledging, in broods in which at least one nestling died, male offspring were significantly more common in years of abundant food—panel C (thick red frame, graphing Model 3 in Table 3). In contrast, in broods with no nestling mortality, male fledglings tended to occur more often in years when food was scarce, but this relationship was not statistically significant—panel D (thin red frame, graphing Model 3 in Table 3). The diameters of points on the tops and bottoms of graphs are proportional to the square root of numbers of offspring at each value along the x‐axes, whose binary response data were modeled.

On average, the sex ratio of hatchlings did not deviate significantly from equality (non‐significant intercepts correspond to a 50% probability that a hatchling will be male, Model 1 in Tables 3 and 4), and we did not find universal patterns of variation in the sex ratio of hatchlings around this average. Instead, our analyses revealed significant differences in sex ratios between early‐ and late‐hatched nestlings in larger broods, but only in broods where at least one egg failed to hatch. This is indicated by the significant interaction between *Hatching order* and egg loss (predictor variable *eLoss*, Model 1 in Table 3): a female‐biased sex ratio dominated among late‐hatched nestlings (negative slope = lower probability of male hatchlings, Figure 2A). While there was a tendency for later‐hatched nestlings to be males in nests without egg loss (Figure 2B), this effect (the main effect of *Hatching order* in Tables 3 and 4) was not statistically significant. Note that the interaction between *Hatching order* and egg loss (*eLoss*) was a statistically significant predictor only when hatching order was an absolute measure (i.e., an ordinal number between 1 and the maximum number of chicks within a brood), but it was not significant when measured as a relative value (i.e., a proportion between 0 for the first hatchling and 1 for the last); contrast in Table 3 and Table 4. The lack of statistical significance in the relative measure (i.e., from 0 to 1; Table 4) suggests that hatching order alone did not influence the sex ratio, but instead, this sex bias in later‐hatched nestlings only occurs in larger broods. Finally, we found no significant effect of inter‐annual variation in *Spring food abundance* or seasonal changes in food (*Spring‐autumn food abundance*) on hatchling sex ratios, nor any significant influence of seasonal variation in *Hatching date*; thus, the results from our analyses do not provide support for our first or second hypotheses.

**TABLE 3:** Results from the modeling variation in: (1) the probability of an offspring being male at hatching, (2) the probability that a nestling will die, (3) the probability of offspring being male at fledging. For continuous predictor variables, a positive regression coefficient indicates an increased probability that a hatchling/fledgling will be male with an increasing value of that predictor variable, or (for nestling mortality) that a nestling will be more likely to die with an increasing value of that predictor. For binary predictor variables, regression coefficients indicate whether there was any difference in the response variable depending on whether any eggs failed to hatch (*eLoss0* describes variation in broods with any egg loss relative to broods without egg loss) or whether there was any mortality of nestlings (*nMort0* describe variation in broods with any nestling mortality relative to broods without nestling mortality). In Model 2, the predictor variable *SexF* describes variation in the mortality of female offspring relative to male offspring. Also in Model 2, the coefficient for *completeSexing0* describes differences in mortality rate for nests in which one or more nestlings died before sexing relative to nests in which all offspring survived until at least the time that blood samples were taken for sexing.

Model	Response variable	Predictor variable	Estimate	Std. error	2.5%	97.5%
1	Sex ratio at hatching	Intercept	0.039	0.128	−0.215	0.295
		eLoss0	−0.012	0.212	−0.397	0.450
		Absolute hatching order	0.158	0.123	−0.063	0.438
		Hatching date	0.198	0.129	−0.049	0.473
		Spring food abundance	−0.148	0.142	−0.441	0.142
		Spring‐autumn food abundance	−0.073	0.129	−0.326	0.192
		eLoss0:Absolute hatching order	**−0.488**	**0.245**	**−0.943**	**−0.023**
		eLoss0:Hatching date	−0.187	0.205	−0.604	0.204
		eLoss0:Spring food abundance	0.212	0.224	−0.179	0.698
		eLoss0:Spring‐autumn food abundance	0.101	0.219	−0.279	0.569
2	Nestling mortality	Intercept	**−6.303**	**1.279**	**−23.967**	**−5.060**
		SexF	0.526	0.739	−1.872	2.881
		Absolute hatching order	**2.955**	**0.728**	**2.188**	**9.522**
		Hatching date	0.693	0.461	−0.207	2.449
		Spring food abundance	**−3.396**	**0.812**	**−10.780**	**−2.588**
		Spring‐autumn food abundance	**−1.463**	**0.616**	**−4.398**	**−0.277**
		completeSexing0	**2.533**	**0.770**	**1.003**	**7.897**
		SexF:Absolute hatching order	−0.524	0.594	−2.369	1.200
		SexF:Hatching date	0.715	0.524	−0.331	3.028
		SexF:Spring food abundance	0.626	0.718	−1.359	2.957
		SexF:Spring‐autumn food abundance	0.080	0.693	−1.839	2.326
3	Sex ratio at fledgling	Intercept	0.152	0.119	−0.070	0.402
		nMort0	0.111	0.214	−0.279	0.589
		Absolute hatching order	0.051	0.117	−0.165	0.285
		Hatching date	0.168	0.113	−0.043	0.408
		Spring food abundance	−0.189	0.143	−0.506	0.091
		Spring‐autumn food abundance	−0.194	0.119	−0.439	0.044
		nMort0:Absolute hatching order	−0.048	0.242	−0.523	0.460
		nMort0:Hatching date	−0.077	0.217	−0.459	0.409
		nMort0:Spring food abundance	**0.692**	**0.230**	**0.331**	**1.204**
		nMort0:Spring‐autumn food abundance	0.372	0.234	−0.029	0.936

*Note:* Statistical significance (bold font) was assessed based on bootstrapped 95% confidence limits of parameter estimates not overlapping zero.

**TABLE 4:** Results from the models examined variations in: (1) the probability of an offspring being male at hatching, (2) the probability that a nestling will die, (3) the probability of offspring being male at fledging. The structure of the models is qualitatively the same as that presented in Table 3 with one difference: *Relative hatching order*, which does not incorporate brood size in the measure, replaced *Absolute hatching order*, which includes information about brood size (Table 3).

Model	Response variable	Predictor variable	Estimate	Std. error	2.5%	97.5%
1	Sex ratio at hatching	Intercept	0.078	0.127	−0.185	0.339
		eLoss0	0.049	0.203	−0.327	0.451
		Relative hatching order	0.132	0.127	−0.114	0.399
		Hatching date	0.191	0.129	−0.076	0.468
		Spring food abundance	−0.094	0.137	−0.363	0.190
		Spring‐autumn food abundance	−0.067	0.129	−0.321	0.191
		eLoss0:Relative hatching order	−0.205	0.203	−0.571	0.186
		eLoss0:Hatching date	−0.182	0.204	−0.594	0.229
		eLoss0:Spring food abundance	0.056	0.213	−0.338	0.462
		eLoss0:Spring‐autumn food abundance	0.078	0.218	−0.305	0.513
2	Nestling mortality	Intercept	**−5.693**	**0.994**	**−28.067**	**−4.618**
		SexF	0.486	0.631	−1.167	2.538
		Relative hatching order	**2.382**	**0.518**	**1.691**	**8.946**
		Hatching date	0.076	0.365	−0.912	1.062
		Spring food abundance	**−1.485**	**0.408**	**−5.989**	**−0.852**
		Spring‐autumn food abundance	−0.849	0.492	−3.122	0.160
		completeSexing0	**3.243**	**0.795**	**2.051**	**10.942**
		SexF:Relative hatching order	−0.376	0.532	−1.917	1.027
		SexF:Hatching date	0.752	0.510	−0.217	3.058
		SexF:Spring food abundance	0.237	0.492	−0.937	2.298
		SexF:Spring‐autumn food abundance	0.045	0.609	−1.697	2.174
3	Sex ratio at fledgling	Intercept	0.135	0.121	−0.085	0.381
		nMort0	0.082	0.226	−0.317	0.573
		Relative hatching order	0.097	0.112	−0.114	0.324
		Hatching date	0.167	0.113	−0.053	0.407
		Spring food abundance	−0.158	0.137	−0.458	0.106
		Spring‐autumn food abundance	−0.187	0.119	−0.436	0.036
		nMort0:Relative hatching order	−0.222	0.241	−0.713	0.310
		nMort0:Hatching date	−0.071	0.216	−0.478	0.378
		nMort0:Spring food abundance	**0.668**	**0.214**	**0.331**	**1.156**
		nMort0:Spring‐autumn food abundance	0.372	0.233	−0.044	0.906

*Note:* Statistical significance (bold font) was assessed based on bootstrapped 95% confidence limits of parameter estimates not overlapping zero.

### 3.2 Nestling Mortality

**FIGURE 3:**
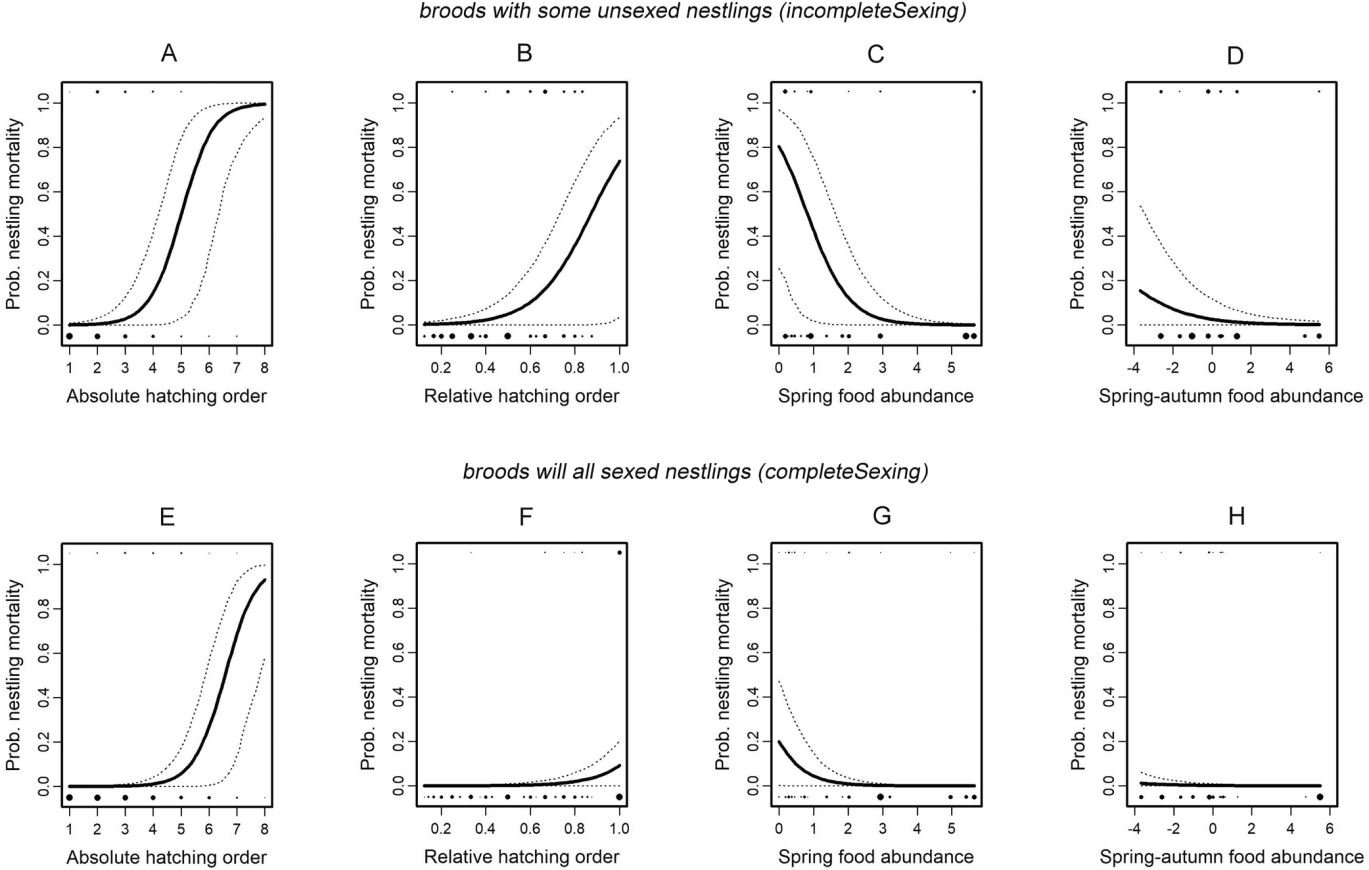
The probability of Boreal Owl nestling mortality varied among years and within nests (Model 2 in Tables 3 and 4). Independent of their sex, later‐hatched nestlings faced significantly higher mortality regardless of whether brood size was measured as the integer rank order of hatching within a brood—panels A and E (graphing Model 2 in Table 3) or as hatch order relative to the first‐hatched and last‐hatched offspring—panels B and F (graphing Model 2 in Table 4). Higher nestling mortality also occurred in years with lower food abundance—panel C and G—and in years with a greater decline in food abundance between spring and autumn—panel D and H (graphing Model 2 in Table 3). The relationships between nestling mortality and significant predictors (panels A–H) were created by modifying the original models, from which we removed the non‐significant predictor offspring *Sex*. Parameter estimates from the displayed relationships in the modified model (Appendix S1) were qualitatively similar to the results of the original models (Tables 3 and 4). The diameter of each dot is proportional to the square root of the numbers of nestlings.

We did not find any statistical evidence of sex‐biased mortality of nestlings because there were no significant interactions between *Sex* and any of the four predictor variables that we hypothesized to be associated with the risk of mortality: *Hatching order*, *Hatching date*, *Spring food abundance*, and within‐year change in food abundance (*Spring‐autumn food abundance*, Model 2 in Tables 3 and 4). Instead, all nestlings, regardless of their sex, were more likely to die if they hatched later (a positive effect of *Hatching order*, Model 2 in Tables 3 and 4, Figures 3A,B,E,F), when food was less abundant in the spring of the nestling season (a negative effect of *Spring food* abundance, Model 2 in Tables 3 and 4, Figures 3C,G), and when food abundance declined from spring to autumn (a negative effect of *Spring‐autumn food abundance*, Model 2 in Table 3, Figures 3D,H). Note that the main effect of *Hatching order* was statistically significant for both absolute measures (Table 3 and Figure 3A,E) and relative measures (Table 4 and Figure 3B,F), suggesting that late‐hatched nestlings were more likely to die than early‐hatched nestlings regardless of the size of the brood. These findings do not provide direct support for our third hypothesis, as neither male nor female nestlings had a significantly higher probability of dying. However, the higher mortality of late‐hatched nestlings may indirectly alter the sex ratio of nestlings if the offspring sex ratio changes with hatching order. In such cases, the sex that is most frequent in late‐hatched nestlings will be the sex most likely to die. Finally, we found no effect of *Hatching date* (Model 2 in Tables 3 and 4), which again did not provide support for our first hypothesis.

The final statistically significant predictor variable in Model 2 describes higher mortality in broods with incomplete sexing (the *completeSexing* predictor in Tables 3 and 4) in which, on average, 1.5 younger nestlings died before being sexed (i.e., typically before nestlings were 14 days old). Unsurprisingly, in these broods, the mortality of nestlings from hatching to fledging was significantly higher than in broods in which all nestlings were sexed. We took this effect into consideration when illustrating the significant sex‐dependent and sex‐independent variations in nestling mortality. Figure 3A–D illustrate the expected patterns for broods with mortality of nestlings before sexing (broods with incomplete sexing of nestlings), while Figure 3E–H illustrate these same patterns for broods with complete sexing. Additionally, in preliminary analyses, we examined whether three‐way interactions were statistically important by adding the completeness of sexing to the original two‐way interactions in Model 2, but none of these three‐way interactions were statistically significant.

### 3.3 Sex Ratio at Fledging

Variation in the sex ratio of fledglings (Model 3, Tables 3 and 4) is the consequence of all adjustments made to offspring sex ratios in previous stages. Consistent with our second hypothesis, annual spring food availability (*Spring food abundance*) was a statistically significant predictor of fledgling sex ratio. However, the pattern was not universal because the main effect of *Spring food abundance* was not statistically significant. Instead, the effect of food abundance was only detected in nests in which some mortality of nestlings (on average, 1.8 nestlings) occurred, as indicated by the significant interaction between *Spring food abundance* and *nMort* (Model 3 in Tables 3 and 4). In these nests, which experienced some nestling mortality, the sex ratios of fledglings were biased toward more males in years with abundant food (positive coefficient in the interaction, Figure 2C). In contrast, in nests without mortality of nestlings, there was only a non‐significant tendency for the opposite pattern of fewer males in high food years (the non‐significant main effect of *Spring food abundance* in Tables 3 and 4 and Figure 2D). No other predictor variables were statistically significant. Thus, there was no effect of within‐season changes in *Spring–autumn food abundance* on fledgling sex ratios. Neither absolute nor relative measurements of *Hatching order* influenced fledgling sex ratios, indicating that the sex bias associated with hatching order (Model 1 in Table 3) did not, on average, translate into the sex ratio of fledglings. Finally, our first hypothesis was again not supported, as variation in *Hatching date* did not significantly affect the fledgling sex ratio (Model 3 in Tables 3 and 4).

## 4 Discussion

Our findings suggest that the sex ratio of Boreal Owl fledglings is shaped by a complex interplay of early maternal adjustments and environmental influences, particularly food abundance, which together determine the sex ratio of fledged offspring. We found no strong evidence of systematic differences in hatchling sex ratios between early‐ and late‐season nests, providing no support for our first hypothesis. Instead, adjustments in fledgling sex ratios were linked to year‐to‐year variations in the abundance of small‐mammal prey, supporting our second hypothesis. However, the role of food abundance was multifaceted, involving multiple mechanisms influencing sex ratios from hatching to fledging:

*At hatching*: Later‐hatched offspring were more likely to be female in larger broods, which were predominantly produced in years of higher food abundance, but only in broods where one or more eggs failed to hatch (Model 1, Figure 2A).

*Post‐hatching selective pressures*: Offspring sex did not directly affect offspring mortality (Model 2). However, the presence of higher mortality of later‐hatched nestlings (Figure 3) means that any biases in sex ratio with hatching order (Model 1) indirectly resulted in sex‐biased mortality of nestlings.

*At fledging*: The offspring sex ratio of fledglings reflected the cumulative effects of all previous adjustments. Together, the result was that more male fledglings were produced in years of high food abundance and large nests, although this bias toward more male fledglings only occurred in nests where one or more nestlings died (Model 3, Figure 2C).

Given the complexity of these patterns, it is unsurprising that published evidence for sex‐ratio adjustments in raptors has been inconsistent (Table 1). This inconsistency highlights the importance of conducting studies across all stages of the nesting cycle to evaluate the process of adjustment of offspring sex ratios. We feel that three aspects of our results, which we will discuss in detail below, are particularly noteworthy:

1. All observed adjustments were associated with offspring loss, which occurred both before and after hatching and was related to inter‐annual food fluctuations.
2. Variation in food abundance influenced sex ratio adjustments through their brood‐size effect, with larger broods exhibiting the most pronounced alterations in sex ratio.
3. Brood size affected whether some adjustments in the sex ratio occurred. This is shown by absolute, but not relative, hatching order being related to a biased sex ratio among hatchlings. In contrast, sex‐independent mortality increased monotonically for later‐hatched nestlings regardless of brood size.

### 4.1 Egg Loss and Nestling Mortality

Our findings revealed that egg loss and nestling mortality caused significant variations in offspring sex ratios. At hatching, the sex ratio was female‐biased for later‐hatched chicks in larger nests where at least one egg did not hatch. In birds, 8%–12% of eggs typically fail to hatch (Marshall et al. 2023), with hatching failure caused by either embryo death or lack of fertilization (Rothstein 1973). In our Boreal Owl population, 16.5% of all laid eggs failed to hatch; egg loss occurred in 50.5% of all clutches, with an average of 1.4 eggs per clutch failing to hatch. Since we did not identify the specific cause of hatching failure, we can only conclude that failure to produce a viable egg—due to either fertilization failure or embryo mortality—is the mechanism behind this adjustment of sex ratio. Further study is needed to determine whether fertilization failure or embryo mortality is the primary cause and which factors trigger these mechanisms (for details, see Love et al. 2005; Assersohn et al. 2021).

Although we did not find direct evidence for sex‐dependent mortality of nestlings (Model 2), sex‐biased mortality of nestlings was an indirect mechanism leading to male‐biased sex ratios in fledglings from the large broods produced during years with high abundance of *Apodemus* mice and *Microtus* voles (Model 3). We believe that the biased sex ratio of fledglings was a result of interactions among hatching order, brood size, food abundance, and changes in food abundance that emerged across the entire nestling cycle. Specifically, at the early nesting stage (at hatching), we observed significant monotonic variation in sex ratio across the hatching sequence, with females becoming more frequent among late‐hatched offspring. This pattern was evident only in broods where some egg loss occurred (the interaction *Hatching order* and *eLoss* in Model 1), suggesting that male‐biased mortality of last‐laid eggs led to a higher proportion of late‐hatched female nestlings in larger broods. Later, at fledging, increased mortality of later‐hatched nestlings, even if statistically unrelated to offspring sex, would indirectly result in more male fledglings in years of high food abundance and large nests, and only in nests where one or more nestlings died (the interaction *Spring food abundance* and *nMort* in Model 3). These results confirm the need to follow Fiala's (1980) caution against assuming the absence of sex‐biased mortality of offspring prior to the determination of nestling sexes.

One of the significant predictors in the model examining nestling mortality (Model 2) was the additional predictor *completeSexing*, which represented the completeness of sexing all hatched nestlings in individual nests. This predictor identified higher mortality in broods where some nestlings were not sexed. In preliminary analyses, we tested three‐way interactions by adding the completeness of sexing to the original two‐way interactions (*Sex* and each continuous predictor in Model 2) and we found that the completeness of sexing did not influence the relationship between sex and the continuous predictors. In summary, the mortality of nestlings in the early part of the nestling period, which is what *completeSexing* describes, is an important part of the overall rate of nestling mortality, but this mortality may not be sex‐biased.

### 4.2 Inter‐Annual Food Fluctuations and Brood Size

Our findings document that substantial year‐to‐year variation in food abundance directly and indirectly served as an important determinant of offspring sex variation in Boreal Owls. This finding is not surprising, as food abundance has effects on various reproductive characteristics in the life history of raptors, such as the Boreal Owl, including clutch size and offspring production (Korpimäki and Hakkarainen 1991; Korpimäki and Hakkarainen 2012; Zárybnická et al. 2015), reproductive strategies (Eldegard and Sonerud 2009, 2010), and fledgling mortality (Kouba et al. 2024). In our study area, the abundance of *Apodemus* mice and *Microtus* voles strongly influences owl reproduction, resulting in the number of fledglings varying from one to eight offspring from a brood. This connection underlines the causal relationship between brood size and food abundance. Specifically, we found that the smaller (less cost) male sex was more frequently represented at fledging in larger broods during years when food was abundant and when any mortality occurred in broods. The tendency to favor the morphologically smaller male fledglings during years of good food availability in our study area aligns with findings from northern regions, including a food supplementation experiment (Hipkiss et al. 2002; Hipkiss and Hörnfeldt 2004). The inconsistency of results with other Boreal Owl studies (Schwerdtfeger and Wink 2014; Kouba et al. 2020) may result from previous studies not assessing adjustments of sex ratio across the entire nestling period, including complex adjustments like we observed in the interplay between brood size and hatching order.

### 4.3 Absolute and Relative Hatching Order

In our study, we described hatching order using two approaches. First, we measured hatching order as absolute values, thus incorporating information about brood size within the measure of hatching order (i.e., the maximum value of hatching order in a nest was determined by the brood size). Second, we used relative values of hatching order, expressed as proportions ranging from 0 (first hatchling) to 1 (last hatchling), which did not contain any information about brood size. We obtained different results from these two ways of describing hatching order. When the sex ratio of hatchlings was the response variable in our models (Model 1, Table 3), absolute hatching order had a significant effect. However, when removing information about brood size by using relative hatching order (Model 1, Table 4), hatching order was not a significant predictor of offspring sex ratio. These results suggest that monotonic changes in offspring sex ratios were dependent upon both brood size and the sequence of hatching. A different effect of hatching order emerged when analyzing nestling mortality (Model 2, Tables 3 and 4), where we found a monotonic increase in mortality for later‐hatched nestlings, independent of brood size. We suggest that both types of hatching order measurements should be considered in future studies, as they offer valuable insights into the mechanisms underlying alterations in sex ratios.

## 5 Conclusions

Past studies of birds such as raptors, with sexual size dimorphism and asynchronous hatching of offspring, have focused on examining the role that differences in offspring body size play in creating unequal sex ratios of offspring (e.g., Torres and Drummond 1997; Hipkiss et al. 2002; Wu et al. 2010). This emphasis carries the implicit assumption that sex‐ratio adjustment through nestling mortality is the primary mechanism for creating sex‐biased offspring ratios at fledging, alongside adjustments in brood size. However, findings in the literature have not consistently supported this assumption, with substantial inconsistencies among studies. Our study revealed that these inconsistencies may arise from a failure to account for the complex mechanisms operating throughout the nesting cycle, driven by an interplay of maternal adjustments and environmental factors, such as food abundance, which directly and indirectly influence sex‐specific mortality through changes in offspring numbers. The limitation of previous studies lies in their focus on investigating variations in offspring sex ratios during short periods or limited phases of the nesting cycle, often with an incomplete set of predictors. We suggest that future studies of sex ratios will need to be based on data from longer‐term studies and across the whole nestling cycle in different environments while simultaneously examining a comprehensive range of potential predictors of offspring sex ratio in order to discover the generality of our findings.

## Author Contributions

**Markéta Zárybnická:** conceptualization (equal), data curation (lead), formal analysis (equal), funding acquisition (equal), investigation (equal), methodology (equal), project administration (lead), visualization (equal), writing – original draft (equal), writing – review and editing (equal). **Lucie Brejšková:** funding acquisition (equal), investigation (equal), methodology (equal), writing – review and editing (supporting). **Karolina Mahlerová:** funding acquisition (equal), investigation (equal), methodology (equal), writing – review and editing (supporting). **Karel Šťastný:** funding acquisition (equal), investigation (equal), writing – review and editing (supporting). **Richard Ševčík:** funding acquisition (equal), investigation (equal), writing – review and editing (supporting). **Fernando Riera:** funding acquisition (equal), investigation (supporting), writing – review and editing (supporting). **Wesley M. Hochachka:** conceptualization (equal), formal analysis (equal), visualization (equal), writing – original draft (equal), writing – review and editing (equal).

## Conflicts of Interest

The authors declare no conflicts of interest.

## Supporting information


Appendix S1.

## Data Availability

The data that support the findings of this study are openly available in the Zenado repository at https://zenodo.org/records/14870612.
