# Supplementary material for: Fledgling Sex Ratio Is Determined by Egg Loss, Hatching Order, Nestling Mortality, and Inter‐Annual Food Fluctuations for Boreal Owls, Aegolius funereus
Source: Ecol Evol. 2025 Mar 13;15(3):e71001. doi: 10.1002/ece3.71001 (PMC11906283; doi:10.1002/ece3.71001)
Supplement: Supplementary file 1 — Appendix S1. [file ECE3-15-e71001-s001.docx]

1. APPENDIX 1

Results from analysis in which (a) the probability of nestling mortality was modeled in relation to *Spring food abundance*, changes in spring-autumn abundance (*Spring-autumn food abundance*), *Hatching date*, and *Absolute hatching order* as in the original model in Table 3, except that the non-significant predictor *Sex* was removed from the model whose results are presented here. The modified analysis was used to create the figures in panels A, C, D, and F in Figure 3. Also presented are results from an analysis in which (b) the probability of nestling mortality was modeled in relation to *Spring food abundance*, *Spring-autumn food abundance*, *Hatching date*, and *Relative hatching order* as in the original model in Table 4, except that the non-significant predictor *Sex* was removed from the model whose results are presented here. This modified analysis was used to create the figures in panels B and E in Figure 3. The results from these modified models are qualitatively similar to those of the original models, in which the predictor *Sex* was included (Tables 3 and 4), with one difference: Seasonal spring-autumn changes in food abundance were a significant predictor in the modified model (a) compared to the original model (Table 3). Statistical significance (bold font) was assessed based on bootstrapped 95% confidence limits of parameter estimates not overlapping zero.

| Response variable | Predictor variable | Estimate | Std. error | 2.5% | 97.5% |
| --- | --- | --- | --- | --- | --- |
| (a) Nestling mortality | Intercept | **−6.027** | **1.311** | **−29.825** | **−4.781** |
|  | Absolute hatching order | **2.639** | **0.626** | **1.982** | **12.483** |
|  | Hatching date | **1.051** | **0.433** | **0.379** | **3.403** |
|  | Spring food abundance | **−3.001** | **0.795** | **−13.237** | **−2.151** |
|  | Spring-autumn food abundance | **−1.147** | **0.501** | **−3.725** | **−0.242** |
|  | completeSexing0 | **2.461** | **0.850** | **0.877** | **7.581** |
| (b) Nestling mortality | Intercept | −5.459 | 0.941 | −27.387 | −4.312 |
|  | Relative hatching order | **2.204** | **0.422** | **1.640** | **8.997** |
|  | Hatching date | 0.412 | 0.297 | −0.167 | 1.918 |
|  | Spring food abundance | **−1.318** | **0.332** | **−4.352** | **−0.756** |
|  | Spring-autumn food abundance | −0.714 | 0.356 | −2.254 | 0.032 |
|  | completeSexing0 | **3.163** | **0.789** | **1.849** | **11.994** |
